# Supplementary material for: HAPDeNovo: a haplotype-based approach for filtering and phasing de novo mutations in linked read sequencing data
Source: BMC Genomics. 2018 Jun 18;19:467. doi: 10.1186/s12864-018-4867-7 (PMC6006847; doi:10.1186/s12864-018-4867-7)
Supplement: Supplementary file 1 — : Table S1 49 de novo mutations for NA12878 (hg19) validated by sanger sequencing. Four gold DNMs (chr5:52638226, chr10:56256293, chr10:56256294, chr20:7195809) could not be evaluated due to poor sequencing coverage of alternative alleles. (PDF 32 kb) [file 12864_2018_4867_MOESM1_ESM.pdf]

Chromosome	Locus	Chromosome	Locus
chr1	75884343	chr10	120325447
chr1	110583335	chr11	40851625
chr1	182974758	chr11	76051551
chr2	39556621	chr11	85631211
chr2	152899032	chr11	117264626
chr2	182693277	chr14	56763353
chr3	101454745	chr14	78809184
chr3	118900031	chr15	50953965
chr4	15227519	chr15	51248561
chr4	104624818	chr15	58669774
chr5	6466106	chr15	85715561
chr5	52638226	chr15	87791919
chr5	126385924	chr15	99175976
chr5	145107247	chr16	29601086
chr6	52120843	chr16	82540504
chr6	145808310	chr17	53502801
chr6	160334960	chr17	71375843
chr8	21568355	chr17	80712655
chr8	74680107	chr18	74117587
chr9	38096405	chr19	6661912
chr9	89775629	chr20	7195809
chr9	123350598	chr20	55356548
chr10	56256293	chr21	20165354
chr10	56256294	chr22	24262476
chrX	8598739		

Table S1: 49 *de novo* mutations for NA12878 (hg19) validated by sanger sequencing. Four gold DNMs (chr5:52638226, chr10:56256293, chr10:56256294, chr20:7195809) could not be evaluated due to poor sequencing coverage of alternative alleles.
