# Supplementary material for: HAPDeNovo: a haplotype-based approach for filtering and phasing de novo mutations in linked read sequencing data
Source: BMC Genomics. 2018 Jun 18;19:467. doi: 10.1186/s12864-018-4867-7 (PMC6006847; doi:10.1186/s12864-018-4867-7)
Supplement: Supplementary file 2 — : Table S2 Comparing the performance between TrioDeNovo and TrioDeNovo+HAPDeNovo with sequencing depth changing from 10 to 30 and different values of DQ. TP (True Positive): the number of DNMs mutations in both candidate set and the gold standard. FP (False Positive): the number of mutations belongs to the candidate set but not in the gold standard. (PDF 42 kb) [file 12864_2018_4867_MOESM2_ESM.pdf]

	Depth	10	11	12	13	14	15	16	17	18	19	20
**TrioDeNovo**	**TP**	44	44	44	44	44	44	43	42	41	39	36
	**FP**	4935	4682	4474	4289	4114	3955	3801	3667	3493	3334	3177
**TrioDeNovo+**	**TP**	44	44	44	44	44	44	43	42	41	39	36
**HAPDeNovo**	**FP**	1036	970	898	848	795	745	695	662	622	585	553
	**Depth**	**21**	**22**	**23**	**24**	**25**	**26**	**27**	**28**	**29**	**30**	
**TrioDeNovo**	**TP**	36	33	33	29	26	25	22	17	13	12	
	**FP**	2997	2759	2545	2353	2110	1888	1694	1468	1277	1095	
**TrioDeNovo+**	**TP**	36	33	33	29	26	25	22	17	13	12	
**HAPDeNovo**	**FP**	521	472	416	383	344	309	278	239	209	175	

Table S2a: Comparing the performance between TrioDeNovo and TrioDeNovo+HAPDeNovo with DQ = 5.

	Depth	10	11	12	13	14	15	16	17	18	19	20
**TrioDeNovo**	**TP**	44	44	44	44	44	44	43	42	41	39	36
	**FP**	4548	4435	4263	4089	3929	3777	3631	3503	3339	3189	3046
**TrioDeNovo+**	**TP**	44	44	44	44	44	44	43	42	41	39	36
**HAPDeNovo**	**FP**	943	903	837	789	742	693	645	612	575	541	511
	**Depth**	**21**	**22**	**23**	**24**	**25**	**26**	**27**	**28**	**29**	**30**	
**TrioDeNovo**	**TP**	36	33	33	29	26	25	22	17	13	12	
	**FP**	2877	2652	2456	2273	2044	1836	1654	1438	1252	1073	
**TrioDeNovo+**	**TP**	36	33	33	29	26	25	22	17	13	12	
**HAPDeNovo**	**FP**	486	441	392	364	329	297	269	233	204	171	

Table S2b: Comparing the performance between TrioDeNovo and TrioDeNovo+HAPDeNovo with DQ = 6.

	Depth	10	11	12	13	14	15	16	17	18	19	20
**TrioDeNovo**	**TP**	44	44	44	44	44	44	43	42	41	39	36
	**FP**	3932	3926	3923	3862	3789	3673	3532	3410	3250	3106	2969
**TrioDeNovo+**	**TP**	44	44	44	44	44	44	43	42	41	39	36
**HAPDeNovo**	**FP**	768	766	765	744	715	674	626	593	558	525	496
	**Depth**	**21**	**22**	**23**	**24**	**25**	**26**	**27**	**28**	**29**	**30**	
**TrioDeNovo**	**TP**	36	33	33	29	26	25	22	17	13	12	
	**FP**	2807	2588	2394	2216	1992	1790	1613	1403	1226	1051	
**TrioDeNovo+**	**TP**	36	33	33	29	26	25	22	17	13	12	
**HAPDeNovo**	**FP**	472	428	379	353	319	289	264	229	201	168	

Table S2c: Comparing the performance between TrioDeNovo and TrioDeNovo+HAPDeNovo with DQ = 7.

	Depth	10	11	12	13	14	15	16	17	18	19	20
**TrioDeNovo**	**TP**	42	42	42	42	42	42	42	41	41	39	36
	**FP**	3373	3368	3366	3362	3360	3355	3350	3282	3189	3049	2913
**TrioDeNovo+**	**TP**	42	42	42	42	42	42	42	41	41	39	36
**HAPDeNovo**	**FP**	592	590	589	589	589	589	587	567	542	510	481
	**Depth**	**21**	**22**	**23**	**24**	**25**	**26**	**27**	**28**	**29**	**30**	
**TrioDeNovo**	**TP**	36	33	33	29	26	25	22	17	13	12	
	**FP**	2751	2536	2343	2167	1948	1750	1576	1372	1196	1023	
**TrioDeNovo+**	**TP**	36	33	33	29	26	25	22	17	13	12	
**HAPDeNovo**	**FP**	457	415	367	342	309	279	254	220	192	160	

Table S2d: Comparing the performance between TrioDeNovo and TrioDeNovo+HAPDeNovo with DQ=8

Table S2: Comparing the performance between TrioDeNovo and TrioDeNovo+HAPDeNovo with sequencing depth changing from 10 to 30 and different values of DQ. **TP** (True Positive): the number of DNMs in both candidate set and the gold standard. **FP** (False Positive): the number of mutations belongs to the candidate set but not in the gold standard.
