# Supplementary material for: HAPDeNovo: a haplotype-based approach for filtering and phasing de novo mutations in linked read sequencing data
Source: BMC Genomics. 2018 Jun 18;19:467. doi: 10.1186/s12864-018-4867-7 (PMC6006847; doi:10.1186/s12864-018-4867-7)
Supplement: Supplementary file 3 — : Table S3 Comparing the performance between FreeBayes and FreeBayes+HAPDeNovo with sequencing depth changing from 10 to 30 and different values of GL. TP (True Positive): the number of DNMs in both candidate set and the gold standard. FP (False Positive): the number of DNMs belongs to the candidate set but not in the gold standard. (PDF 43 kb) [file 12864_2018_4867_MOESM3_ESM.pdf]

	Depth	10	11	12	13	14	15	16	17	18	19	20
**FreeBayes**	**TP**	44	44	44	44	44	44	43	42	41	39	36
	**FP**	15796	14477	13385	12279	11280	10387	9507	8789	8069	7378	6744
**FreeBayes+**	**TP**	44	44	44	44	44	44	43	42	41	39	36
**HAPDeNovo**	**FP**	3724	3401	3090	2794	2557	2312	2062	1876	1687	1500	1345
	**Depth**	**21**	**22**	**23**	**24**	**25**	**26**	**27**	**28**	**29**	**30**	
**FreeBayes**	**TP**	36	33	33	29	26	25	22	17	13	12	
	**FP**	6122	5526	4970	4469	3985	3579	3206	2811	2463	2127	
**FreeBayes+**	**TP**	36	33	33	29	26	25	22	17	13	12	
**HAPDeNovo**	**FP**	1194	1070	929	823	731	662	591	512	441	377	

Table S3a: Comparing the performance between FreeBayes and FreeBayes+HAPDeNovo without GL threshold.

	Depth	10	11	12	13	14	15	16	17	18	19	20
**FreeBayes**	**TP**	44	44	44	44	44	44	43	42	41	39	36
	**FP**	5902	5902	5902	5902	5902	5785	5573	5342	5068	4771	4414
**FreeBayes+**	**TP**	44	44	44	44	44	44	43	42	41	39	36
**HAPDeNovo**	**FP**	1115	1115	1115	1115	1115	1083	1025	970	908	839	765
	**Depth**	**21**	**22**	**23**	**24**	**25**	**26**	**27**	**28**	**29**	**30**	
**FreeBayes**	**TP**	36	33	33	29	26	25	22	17	13	12	
	**FP**	4053	3674	3298	2992	2671	2390	2134	1855	1619	1398	
**FreeBayes+**	**TP**	36	33	33	29	26	25	22	17	13	12	
**HAPDeNovo**	**FP**	689	616	533	479	427	385	345	296	254	216	

Table S3b: Comparing the performance between FreeBayes and FreeBayes+HAPDeNovo with GL = -50.

	Depth	10	11	12	13	14	15	16	17	18	19	20
**FreeBayes**	**TP**	39	39	39	39	39	39	39	39	38	36	34
	**FP**	4920	4920	4920	4920	4920	4920	4920	4903	4758	4553	4285
**FreeBayes+**	**TP**	39	39	39	39	39	39	39	39	38	36	34
**HAPDeNovo**	**FP**	874	874	874	874	874	874	874	868	836	789	734
	**Depth**	**21**	**22**	**23**	**24**	**25**	**26**	**27**	**28**	**29**	**30**	
**FreeBayes**	**TP**	34	32	32	28	25	24	22	17	13	12	
	**FP**	3979	3630	3276	2982	2666	2388	2134	1855	1619	1398	
**FreeBayes+**	**TP**	34	32	32	28	25	24	22	17	13	12	
**HAPDeNovo**	**FP**	672	606	527	475	426	385	345	296	254	216	

Table S3c: Comparing the performance between FreeBayes and FreeBayes+HAPDeNovo with GL = -60

Table S3: Comparing the performance between FreeBayes and FreeBayes+HAPDeNovo with sequencing depth changing from 10 to 30 and different values of GL. **TP** (True Positive): the number of DNMs in both candidate set and the gold standard. **FP** (False Positive): the number of DNMs belongs to the candidate set but not in the gold standard.
