# Supplementary material for: HAPDeNovo: a haplotype-based approach for filtering and phasing de novo mutations in linked read sequencing data
Source: BMC Genomics. 2018 Jun 18;19:467. doi: 10.1186/s12864-018-4867-7 (PMC6006847; doi:10.1186/s12864-018-4867-7)
Supplement: Supplementary file 4 — : Table S4 Comparing the performance between GATK and GATK+HAPDeNovo with sequencing depth changing from 10 to 30 and with different values of PL. TP (True Positive): the number of DNMs in both candidate set and the gold standard. FP (False Positive): the number of DNMs belongs to the candidate set but not in the gold standard. (PDF 42 kb) [file 12864_2018_4867_MOESM4_ESM.pdf]

	**Depth**	**10**	**11**	**12**	**13**	**14**	**15**	**16**	**17**	**18**	**19**	**20**
**GATK**	**TP**	43	43	43	43	43	43	42	42	41	39	37
	**FP**	271542	268864	266013	263122	259699	255737	251139	245615	239464	232340	224186
**GATK+**	**TP**	43	43	43	43	43	43	42	42	41	39	37
**HAPDeNovo**	**FP**	2599	2418	2236	2054	1905	1721	1578	1421	1290	1177	1072
	**Depth**	**21**	**22**	**23**	**24**	**25**	**26**	**27**	**28**	**29**	**30**	
**GATK**	**TP**	36	36	33	31	30	25	19	19	15	14	
	**FP**	214883	204326	193291	181810	169297	156400	143602	130712	117970	105360	
**GATK+**	**TP**	36	36	33	31	30	25	19	19	15	14	
**HAPDeNovo**	**FP**	978	861	762	680	605	529	480	422	372	322	

Table S4a: Comparing the performance between GATK and GATK+HAPDeNovo without PL threshold

	**Depth**	**10**	**11**	**12**	**13**	**14**	**15**	**16**	**17**	**18**	**19**	**20**
**GATK**	**TP**	43	43	43	43	43	43	42	42	41	39	37
	**FP**	247016	246812	246362	245617	244372	242487	239654	235914	231399	225706	218900
**GATK+**	**TP**	43	43	43	43	43	43	42	42	41	39	37
**HAPDeNovo**	**FP**	1592	1580	1560	1526	1481	1396	1315	1237	1148	1060	983
	**Depth**	**21**	**22**	**23**	**24**	**25**	**26**	**27**	**28**	**29**	**30**	
**GATK**	**TP**	36	36	33	31	30	25	19	19	15	14	
	**FP**	210742	201060	190734	179797	167704	155112	142568	129865	117269	104773	
**GATK+**	**TP**	36	36	33	31	30	25	19	19	15	14	
**HAPDeNovo**	**FP**	905	809	725	654	589	519	472	417	368	320	

Table S4b: Comparing the performance between GATK and GATK+HAPDeNovo with PL = 450.

	**Depth**	**10**	**11**	**12**	**13**	**14**	**15**	**16**	**17**	**18**	**19**	**20**
**GATK**	**TP**	41	41	41	41	41	41	40	40	39	38	36
	**FP**	240698	240600	240482	240150	239352	238010	235681	232348	228224	222917	216593
**GATK+**	**TP**	41	41	41	41	41	41	40	40	39	38	36
**HAPDeNovo**	**FP**	1377	1375	1370	1357	1336	1286	1233	1171	1094	1017	950
	**Depth**	**21**	**22**	**23**	**24**	**25**	**26**	**27**	**28**	**29**	**30**	
**GATK**	**TP**	35	35	32	30	29	24	19	19	15	14	
	**FP**	208861	199612	189604	178932	167041	154585	142152	129559	117042	104592	
**GATK+**	**TP**	35	35	32	30	29	24	19	19	15	14	
**HAPDeNovo**	**FP**	879	793	712	646	582	513	467	415	366	318	

Table S4c: Comparing the performance between GATK and GATK+HAPDeNovo with PL = 500.

Table S4: Comparing the performance between GATK and GATK+HAPDeNovo with sequencing depth changing from 10 to 30 and with different values of PL. **TP** (True Positive): the number of DNMs in both candidate set and the gold standard. **FP** (False Positive): the number of DNMs belongs to the candidate set but not in the gold standard.
