# Supplementary material for: HAPDeNovo: a haplotype-based approach for filtering and phasing de novo mutations in linked read sequencing data
Source: BMC Genomics. 2018 Jun 18;19:467. doi: 10.1186/s12864-018-4867-7 (PMC6006847; doi:10.1186/s12864-018-4867-7)
Supplement: Supplementary file 5 — : Table S5 Comparing the performance between DeNovoGear and DeNovoGear+HAPDeNovo with sequencing depth changing from 10 to 30 and with different values of PP. TP (True Positive): the number of DNMs in both candidate set and the gold standard. FP (False Positive): the number of DNMs belongs to the candidate set but not in the gold standard. (PDF 42 kb) [file 12864_2018_4867_MOESM5_ESM.pdf]

	**Depth**	**10**	**11**	**12**	**13**	**14**	**15**	**16**	**17**	**18**	**19**	**20**
**DeNovoGear**	**TP**	40	40	40	40	40	40	39	39	38	36	35
	**FP**	44346	44346	43597	43169	42679	42097	41391	40535	39301	37801	36252
**DeNovoGear+**	**TP**	40	40	40	40	40	40	39	39	38	36	35
**HAPDeNovo**	**FP**	999	953	913	868	834	793	755	713	664	610	560
	**Depth**	**21**	**22**	**23**	**24**	**25**	**26**	**27**	**28**	**29**	**30**	
**DeNovoGear**	**TP**	34	34	31	29	28	23	18	18	14	13	
	**FP**	34472	32464	30303	28194	26070	23861	21791	19774	17838	15993	
**DeNovoGear+**	**TP**	34	34	31	29	28	23	18	18	14	13	
**HAPDeNovo**	**FP**	524	470	419	377	347	319	291	257	230	201	

Table S5a: Comparing the performance between DeNovoGear and DeNovoGear+HAPDeNovo with PP = 1E-4.

	**Depth**	**10**	**11**	**12**	**13**	**14**	**15**	**16**	**17**	**18**	**19**	**20**
**DeNovoGear**	**TP**	43	43	43	43	43	43	42	42	41	39	37
	**FP**	94580	93642	92705	91702	90544	89187	87553	85588	83475	80722	77634
**DeNovoGear+**	**TP**	43	43	43	43	43	43	42	42	41	39	37
**HAPDeNovo**	**FP**	1808	1693	1572	1448	1360	1241	1154	1058	979	902	832
	**Depth**	**21**	**22**	**23**	**24**	**25**	**26**	**27**	**28**	**29**	**30**	
**DeNovoGear**	**TP**	36	36	33	31	30	25	19	19	15	14	
	**FP**	74078	70162	65982	61727	57156	52466	48007	43538	39312	35219	
**DeNovoGear+**	**TP**	36	36	33	31	30	25	19	19	15	14	
**HAPDeNovo**	**FP**	768	687	618	548	498	443	403	356	316	273	

Table S 5b: Comparing the performance between DeNovoGear and DeNovoGear+HAPDeNovo with PP = 3E-5.

	**Depth**	**10**	**11**	**12**	**13**	**14**	**15**	**16**	**17**	**18**	**19**	**20**
**DeNovoGear**	**TP**	40	40	40	40	40	40	39	39	38	36	35
	**FP**	46804	46401	45997	45547	45027	44415	43610	42447	41132	39587	37993
**DeNovoGear+**	**TP**	40	40	40	40	40	40	39	39	38	36	35
**HAPDeNovo**	**FP**	1060	1011	970	920	883	842	799	742	688	633	582
	**Depth**	**21**	**22**	**23**	**24**	**25**	**26**	**27**	**28**	**29**	**30**	
**DeNovoGear**	**TP**	34	34	31	29	28	23	18	18	14	13	
	**FP**	36151	34058	31836	29649	27446	25145	23005	20896	18853	16901	
**DeNovoGear+**	**TP**	34	34	31	29	28	23	18	18	14	13	
**HAPDeNovo**	**FP**	543	484	433	389	357	328	300	265	236	207	

Table S5c: Comparing the performance between DeNovoGear and DeNovoGear+HAPDeNovo with PP = 5E-5.

Table S5: Comparing the performance between DeNovoGear and DeNovoGear+HAPDeNovo with sequencing depth changing from 10 to 30 and with different values of PP. **TP** (True Positive): the number of DNMs in both candidate set and the gold standard. **FP** (False Positive): the number of DNMs belongs to the candidate set but not in the gold standard.
