# Supplementary material for: HAPDeNovo: a haplotype-based approach for filtering and phasing de novo mutations in linked read sequencing data
Source: BMC Genomics. 2018 Jun 18;19:467. doi: 10.1186/s12864-018-4867-7 (PMC6006847; doi:10.1186/s12864-018-4867-7)
Supplement: Supplementary file 6 — : Table S6. Comparing the performance only on X chromosome for FreeBayes, TrioDenovo, GATK, and DeNovoGear before and after applying HAPDeNovo. (PDF 44 kb) [file 12864_2018_4867_MOESM6_ESM.pdf]

| | Depth | 10 | 11 | 12 | 13 | 14 | 15 | 16 | 17 | 18 | 19 | 20 |
|---|---|---|---|---|---|---|---|---|---|---|---|---|
| **FreeBayes** | **TP** | 1 | 1 | 1 | 1 | 1 | 1 | 0 | 0 | 0 | 0 | 0 |
| | **FP** | 272 | 272 | 272 | 272 | 272 | 245 | 213 | 182 | 154 | 123 | 103 |
| **FreeBayes+** | **TP** | 1 | 1 | 1 | 1 | 1 | 1 | 0 | 0 | 0 | 0 | 0 |
| **HAPDeNovo** | **FP** | 44 | 44 | 44 | 44 | 44 | 39 | 30 | 27 | 18 | 15 | 11 |
| **TrioDeNovo** | **TP** | 1 | 1 | 1 | 1 | 1 | 1 | 0 | 0 | 0 | 0 | 0 |
| | **FP** | 248 | 248 | 248 | 240 | 229 | 198 | 168 | 145 | 120 | 97 | 78 |
| **TrioDeNovo+** | **TP** | 1 | 1 | 1 | 1 | 1 | 1 | 0 | 0 | 0 | 0 | 0 |
| **HAPDeNovo** | **FP** | 38 | 38 | 38 | 37 | 36 | 30 | 23 | 22 | 14 | 13 | 9 |
| **DeNovoGear** | **TP** | 0 | 0 | 0 | 0 | 0 | 0 | 0 | 0 | 0 | 0 | 0 |
| | **FP** | 24 | 24 | 23 | 23 | 23 | 22 | 21 | 21 | 20 | 20 | 19 |
| **DeNovoGear+** | **TP** | 0 | 0 | 0 | 0 | 0 | 0 | 0 | 0 | 0 | 0 | 0 |
| **HAPDeNovo** | **FP** | 1 | 1 | 0 | 0 | 0 | 0 | 0 | 0 | 0 | 0 | 0 |

Table S6a: Comparing the performance between TrioDeNovo and TrioDeNovo+HAPDeNovo with DQ = 7, and between FreeBayes and FreeBayes+HAPDeNovo with GL = -50, with sequencing depth changing from 10 to 20.

| | Depth | 35 | 36 | 37 | 38 | 39 | 40 | 41 | 42 | 43 | 44 | 45 |
|---|---|---|---|---|---|---|---|---|---|---|---|---|
| **GATK** | **TP** | 1 | 1 | 1 | 1 | 1 | 0 | 0 | 0 | 0 | 0 | 0 |
| | **FP** | 12059 | 10465 | 9029 | 7690 | 6605 | 5529 | 4614 | 3916 | 3244 | 2726 | 2279 |
| **GATK+** | **TP** | 1 | 1 | 1 | 1 | 1 | 0 | 0 | 0 | 0 | 0 | 0 |
| **HAPDeNovo** | **FP** | 11 | 9 | 9 | 8 | 5 | 5 | 4 | 4 | 4 | 3 | 3 |

Table S6b: Comparing the performance between GATK and GATK+HAPDeNovo with PL = 450 with sequencing depth changing from 35 to 45.

| | Depth | 1 | 2 | 3 | 4 | 5 | 6 | 7 | 8 | 9 | 10 |
|---|---|---|---|---|---|---|---|---|---|---|---|
| **DeNovoGear** | **TP** | 0 | 0 | 0 | 0 | 0 | 0 | 0 | 0 | 0 | 0 |
| | **FP** | 251 | 144 | 87 | 53 | 44 | 34 | 31 | 28 | 26 | 24 |
| **DeNovoGear+** | **TP** | 0 | 0 | 0 | 0 | 0 | 0 | 0 | 0 | 0 | 0 |
| **HAPDeNovo** | **FP** | 65 | 33 | 18 | 7 | 5 | 3 | 3 | 1 | 1 | 1 |

Table S6c: Comparing the performance between DeNovoGear and DeNovoGear+HAPDeNovo with PP = 3E-5 with sequencing depth changing from 1 to 10.

Table S6: Comparing the performance only on X chromosome for FreeBayes, TrioDeNovo, GATK, and DeNovoGear before and after applying HAPDeNovo. **TP** (True Positive): the number of DNMs in both candidate set and the gold standard. **FP** (False Positive): the number of DNMs belongs to the candidate set but not in the gold standard.
